# Identification of metabolic phenotypes in childhood obesity by ^1^H NMR metabolomics of blood plasma

**DOI:** 10.4155/fsoa-2017-0146

**Published:** 2018-05-23

**Authors:** Liene Bervoets, Guy Massa, Wanda Guedens, Gunter Reekmans, Jean-Paul Noben, Peter Adriaensens

**Affiliations:** 1 Faculty of Medicine & Life Sciences, Hasselt University, Martelarenlaan 42, 3500 Hasselt, Belgium; 2 Department of Pediatrics, Jessa Hospital, Stadsomvaart 11, 3500 Hasselt, Belgium; 3 Biomolecule Design Group, Institute for Materials Research, Hasselt University, Agoralaan Building D, 3590 Diepenbeek, Belgium; 4 Applied & Analytical Chemistry, Institute for Materials Research, Hasselt University, Agoralaan Building D, 3590 Diepenbeek, Belgium; 5 Biomedical Research Institute, Hasselt University, Agoralaan Building C, 3590 Diepenbeek, Belgium

**Keywords:** amino acids, childhood obesity, metabolically healthy obesity, metabolic syndrome, metabolomics, multivariate analysis, N-acetyl glycoproteins, NMR spectroscopy, obesity, phospholipids

## Abstract

**Aim:**

To identify the plasma metabolic profile associated with childhood obesity and its metabolic phenotypes.

**Materials & methods:**

The plasma metabolic profile of 65 obese and 37 normal-weight children was obtained using proton NMR spectroscopy. NMR spectra were rationally divided into 110 integration regions, which reflect relative metabolite concentrations, and were used as statistical variables.

**Results:**

Obese children show increased levels of lipids, N-acetyl glycoproteins, and lactate, and decreased levels of several amino acids, α-ketoglutarate, glucose, citrate, and cholinated phospholipids as compared with normal-weight children. Metabolically healthy children show lower levels of lipids and lactate, and higher levels of several amino acids and cholinated phospholipids, as compared with unhealthy children.

**Conclusion:**

This study reveals new valuable findings in the field of metabolomics and childhood obesity. Although validation should be performed, the proof of principle looks promising and justifies a deeper investigation of the diagnostic possibilities of proton NMR metabolomics in follow-up studies.

**Trial registration:** NCT03014856. Registered January 9, 2017.

## Lay abstract

The plasma metabolic profile of childhood obesity and its metabolic phenotypes was identified using untargeted proton NMR spectroscopy combined with multivariate statistics. Obese children show increased plasma levels of lipids, N-acetyl glycoproteins and lactate, next to decreased levels of several amino acids, α-ketoglutarate, glucose, citrate and cholinated phospholipids as compared with normal-weight children. In addition, the metabolic profile of healthy and unhealthy obese children could be discriminated and although further validation should be performed, these findings might pave the way to a detailed diagnostic metabolic signature in children.

## Graphical abstract

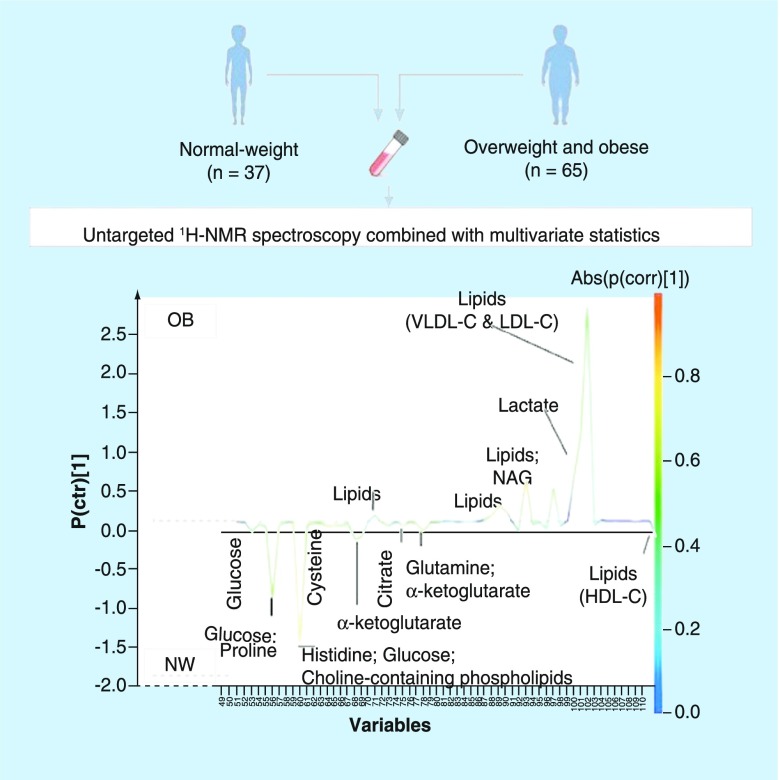

Childhood obesity has become a major public health crisis with high prevalence numbers worldwide [1]. Obese  children and adolescents are at high risk to develop metabolic disorders such as insulin resistance, metabolic syndrome, Type 2 diabetes mellitus, coronary artery diseases and nonalcoholic fatty liver disease [2]. A majority of obese children will also become obese as an adult [3]. Although progress is being made to combat childhood obesity, it is of utmost importance to develop effective and targeted prevention and treatment strategies [4]. Hereto, the concept of metabolically healthy obesity (MHO) was recently introduced. MHO children are obese, but do not show any metabolic complication [5,6]. Early screening for MHO might help to refocus current prevention and treatment strategies, and may even result in a reduction of healthcare costs [6]. However, a good understanding of obesity-related biochemical mechanisms is inevitable for the development of appropriate screening strategies.

In recent years, human-based metabolomics has emerged into a powerful high-throughput tool to identify and quantify metabolites in biofluids such as plasma and urine [7]. The so-called metabolome provides a direct read-out of an organism's clinical phenotype, an indicator of both genetic and environmental perturbations [8]. Consequently, the interest in metabolomics as a platform to study the underlying biochemical mechanisms of obesity and related metabolic disorders is increasing [9]. Up to now, metabolomics studies focusing on obesity in childhood have been performed only with mass spectrometry as analytical tool and show some conflicting results [10–13]. More recently, high resolution ^1^H NMR spectroscopy has proven to be a robust technique to detect disease and to investigate disease mechanisms [7,14,15], but has been rarely applied as metabolomic profiling tool to investigate biochemical pathways associated with childhood obesity [9]. NMR spectroscopy offers excellent stability and integration accuracy (linear response between metabolite concentration and signal intensity/integration value), allowing not only to identify, but also to quantify the metabolites in biological samples. Moreover, since there is no need for solvent extractions of the sample, the reproducibility is very high [16,17].

The main objective of this study was to investigate and compare plasma metabolic profiles of obese and normal-weight children using an untargeted ^1^H NMR-based metabolomics approach. In addition, the plasma metabolic profiles of MHO and metabolically unhealthy obese (MUO) children and adolescents were examined and compared, something that has – to the best of our knowledge – not been considered before.

## Materials & methods

### Study population

The study population (Table 1) consists of 65 OB (8 overweight and 57 obese) children and 37 NW (normal-weight) children and adolescents aged between 8 and 18 years (Clinical Trial Registration: NCT03014856. Registered 9 January 2017). The 65 OB children underwent an oral glucose tolerance test as part of a multidisciplinary obesity evaluation and were recruited at the outpatient pediatric obesity clinic of the Jessa Hospital (Hasselt, Belgium) between February 2012 and December 2013. The 37 NW children were recruited among the children of personnel working at the Jessa Hospital or Hasselt University (Hasselt, Belgium). Inclusion criteria were: aged 8–18 years; normal-weight, overweight or obese according to the International Obesity Task Force (IOTF) BMI criteria [18]; fasted for at least 8 h. Weight status was classified according to the IOTF criteria and not based on a measurement of absolute fat mass or percentage of total body fat. Indirect bioelectrical impedance analysis was measured; however, there is still no single bioelectrical impedance analysis equation that can be applied for both children and adolescents because of pubertal influences resulting in poor accuracy. Subjects taking any medication or having serious chronic or acute illness within 2 weeks preceding the clinical examination were excluded from the study. All patients were of Caucasian origin. The study was conducted in accordance with the ethical rules of the Helsinki Declaration and Good Clinical Practice. The study protocol was approved by the medical-ethical committees of the Jessa Hospital and Hasselt University (approval number: 12.27/ped12.02/OBNMR1). Informed and written consent was obtained from all participants and their parents or legal guardian.

**Table 1.** **Descriptive and clinical characteristics of the studied individuals.**

**Descriptive characteristics**	**OB (overweight, n = 8; obese, n = 57)^†^**	**NW (n = 37)**	**p-value**
Age, years	13.1 ± 2.2	13.0 ± 2.7	0.798

Gender, n (%) Male Female	39 (60.0) 26 (40.0)	18 (48.6) 19 (51.4)	0.267

Pubertal stage^‡^, n (%) I (prepubertal) II–IV (pubertal) V (postpubertal)	14 (21.5) 26 (40.0) 21 (32.3)	11 (29.7) 13 (35.1) 9 (24.3)	0.531

Weight, kg	83.1 ± 21.8	46.3 ± 11.6	<0.001

Height, cm	160.9 ± 11.8	157.6 ± 13.1	0.197

BMI, kg/m^2^	32.0 (18.5–44.1)	18.0 (15.1–23.8)	<0.001

BMI SDS	2.8 (1.2–3.7)	-0.1 (-1.1–1.2)	<0.001

SBP, mm Hg	122 ± 11	115 ± 11	0.001

DBP, mm Hg	74 ± 8	69 ± 8	0.004

Continuous variables are presented as mean ± SD or median (range) and nominal or ordinal scale variables as a number with percentage (%).

^†^According to the IOTF criteria (18).

^‡^Eight participants refused clinical examination to determine pubertal stage.

DBP: Diastolic blood pressure; IOTF: International Obesity Task Force; NW: Normal-weight; OB: Overweight or obese; SBP: Systolic blood pressure; SDS: Standard deviation score.

### Anthropometry

The pubertal developmental stage was determined according to Tanner by clinical examination or self-assessment using realistic color images [19,20]. Tanner stage was determined on the basis of breast development and genital size and was categorized into three groups: prepubertal (Tanner stage I), pubertal (Tanner stage II–IV) and postpubertal (Tanner stage V). Standing height was measured to the nearest 0.1 cm using a Harpenden wall stadiometer, and weight was measured to the nearest 0.1 kg using an electronic balance scale. BMI was calculated by dividing weight in kilograms by height in meters squared (BMI = kg/m^2^). BMI standard deviation score (SDS) was calculated by the LMS method based on the IOTF criteria recently proposed by Cole *et al*. [18]. Seated blood pressure was measured twice with an electronic sphygmomanometer (Omron^®^, Omron Healthcare, IL, USA) according to a validated protocol and the two measurements were averaged [21].

### Conventional biochemical analyses

Biochemical laboratory parameters were only assessed in the study group with obesity. After an overnight fast, venous blood samples were taken for the analysis of the following biochemical parameters: glucose, high-density lipoprotein cholesterol (HDL-C) and triglycerides. Plasma glucose was measured by the glucose oxidase method using a Synchron LX20 analyzer (Beckman Coulter, CA, USA). Plasma HDL-C and triglycerides were measured on a Beckman Coulter AU 2700 automatic analyzer. Total cholesterol, low-density lipoprotein (LDL)-cholesterol, hemoglobin A1c, and uric acid were also measured, but they were not included in the definition of MHO (see below). Also sex hormones, such as sex hormone binding globulin and testosterone, were measured, but data were incomplete and therefore not included in further analyses.

### Metabolically healthy obesity

MHO individuals are classified as being obese (according to IOTF criteria) but having none of the components of the metabolic syndrome [5]. The metabolic syndrome was defined according to the criteria of the International Diabetes Federation consensus for children older than 10 years [22]. Apart from obesity, the metabolic syndrome is defined as having two or more of the following abnormalities: HDL-C <0 mg/dl (females 16 years or older: HDL-C <50 mg/dl); triglycerides ≥ 150 mg/dl; systolic blood pressure (SBP) ≥ 130 mm Hg or diastolic blood pressure (DBP) ≥ 85 mm Hg; fasting plasma glucose ≥ 100 mg/dl. MUO subjects are classified as being obese and having two or more components of the metabolic syndrome. A group that fell in-between the classification of MHO and MUO was termed metabolically at-risk obese, but this group was not included in the statistical analyses.

#### Sample collection, sample preparation & ^1^H NMR spectroscopy

Fasting venous blood was collected in 6 ml lithium heparin tubes and stored at 4°C within 10 min. Within 30 min of collection, samples were centrifuged at 1600 g for 15 min and plasma aliquots of 500 μl were transferred into cryovials and stored at -80°C [23]. Detailed protocols regarding sample preparation and ^1^H NMR analysis have been previously described [14,24]. ^1^H NMR spectra were rationally divided into 110 integration regions, defined on the basis of spiking experiments with known metabolites [25]. These integration regions reflect the relative metabolite concentrations, in other words, the metabolic phenotype, and were used as statistical variables to construct (train) a statistical classification model in discriminating between OB and NW, and between MHO and MUO. Some of the described methodology is similar to Bervoets *et al*. [26].

### Statistical analyses

Differences between cases and controls were examined using univariate analyses (IBM SPSS version 20.0, SPSS, Inc., IL, USA). The distribution of the data was tested for normality using the Kolmogorov–Smirnov test, and visual assessment of Q-Q plots. Normally distributed continuous variables were tested with the independent samples t test and results are presented as mean ± SD. Non-normally distributed continuous variables were tested with the Mann–Whitney *U* test and results are presented as median (range). Chi-square test of association (n ≥10) or Fisher exact probability test (n < 10) was applied to test nominal or ordinal variables between two or more groups. For 3 × 2 contingency tables with n < 10, the Freeman–Halton extension of the Fisher exact probability test was used. Nominal scale variables (variables with values representing two or more categories without having any kind of natural order, such as gender) and ordinal scale variables (variables with values representing two or more categories that are clearly ordered, such as puberty) are presented as a number with percentage (%). All p-values smaller than 0.05 are considered significant. Multivariate statistics was performed using SIMCA-P^+^ (Version 13.0, Umetrics, Sweden). After mean-centering and Pareto scaling of the variables, unsupervised principal component analysis (PCA) was performed in order to look for clustering and possible confounders within the dataset, and to identify possible outliers by using a Hotelling's T^2^ range test and a distance to model plot. In the next step, orthogonal partial least squares discriminant analysis (OPLS-DA) was used to build (train) a model (statistical classifier) to discriminate between OB and NW, and between MHO and MUO [27]. The validity of the established models was evaluated on one hand by the total amount of variation between and within the groups that is explained by the model [denoted as R^2^Y(cum) and R^2^X(cum), respectively] and on the other hand by the predictive ability of the model as determined by a sevenfold cross-validation [denoted as Q^2^(cum)]. Levels of sensitivity (the percentage of OB or MUO that are actually classified as OB or MUO) and specificity (the percentage of NW or MHO that are actually classified as NW or MHO) were calculated in order to determine how well the OPLS-DA model classifies the observations into known classes. To be defined as a variable that strongly contributes to the group discrimination, three selection criteria have to be fulfilled: significantly different in univariate statistics (a student t-test corrected for multiple testing by the Benjamini–Hochberg method); an OPLS-DA absolute value of p(corr), in other words, the loading scaled as a correlation coefficient, exceeding 0.5; and an OPLS-DA variable importance for the projection (VIP) value exceeding 0.7 [27].

## Results

### Plasma metabolic profile of OB versus NW children & adolescents

The descriptive and clinical characteristics of the study population are presented in Table 1. The study consists of 65 OB (eight overweight and 57 obese) and 37 NW children and adolescents aged between 8 and 18 years (mean age: 13.1 ± 2.4 years). OB and NW children do not differ significantly in terms of age, gender, and pubertal stage. Weight, BMI, BMI SDS, SBP and DBP are significantly higher in OB as compared with NW children and adolescents. A zoom-in of a typical ^1^H NMR spectrum of OB and NW children is illustrated in Supplementary Figure 1.

**Figure 1.**
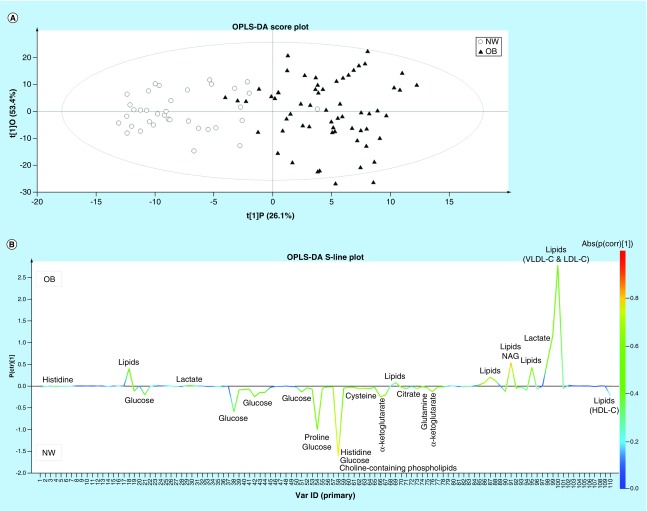
**Discrimination between OB and NW based on the ^1^H-NMR-derived metabolic phenotype of blood plasma.** OPLS-DA score plot **(A)** and S-line plot **(B)** obtained for the OB (▴) and NW (○) children and adolescents. Each participant is represented by its metabolic profile and visualized as a single symbol of which the location is determined by the contributions of the 110 variables in the ^1^H NMR spectrum. The OPLS-DA score plot (LV = 3) shows the first predictive component (t[1]P: 26.1%), explaining the variation between the groups, versus the first orthogonal component (t[1]O: 53.4%) that explains the variation within the groups. The OPLS-DA S-line plot visualizes differences between OB patients (positive) and NW controls (negative). The left y-axis represents p(ctr)[1], the covariance between a variable and the classification score. It indicates if an increase or decrease of a variable is correlated to the classification score. The magnitude of the covariance is however difficult to interpret since covariance is scale dependent. This means that a high value for the covariance does not necessary imply a strong correlation, as the covariance is also influenced by the intensity of the signal with respect to the noise level. Therefore this measure will likely indicate variables with large signal intensities. On the right y-axis, p(corr)[1] is the correlation coefficient between a variable and the classification score, for example, the normalized covariance. It gives a linear indication of the strength of the correlation. As the correlation is independent of the intensity of the variable, it will be a better measure for the reliability of the variable in the classification process. In Figure 1B, the red color stands for the highest absolute value of the correlation coefficient. Strongly discriminating variables have a large intensity and large reliability. NW: Normal-weight; OB: Overweight or obese; OPLS-DA: Orthogonal partial least square discriminant analysis.

For the metabolic profiling, multivariate OPLS-DA statistics was used to train a classification model (classifier) in discriminating between OB patients and NW controls based on data input from their metabolic profile, in other words, the integration values of the 110 regions in the ^1^H NMR spectra or variables. A PCA analysis was however conducted first to look for clustering, possible confounders and outliers. Five outliers (four OB and one NW) were detected and subsequently removed from the model, resulting in a final dataset of 61 OB and 36 NW individuals. The PCA score plots illustrate that individuals, each represented by the values of the 110 variables obtained from its ^1^H NMR spectrum (its metabolic profile), are clustered in a way that allows OB individuals to be clearly differentiated from their NW counterparts (Supplementary Figure 2). The PCA score plots show no clustering of observations when colored according to age, gender or pubertal status, indicating that these variables are no confounders (Supplementary Figure 2). Multivariate OPLS-DA was used to train a classification model (classifier) in discriminating between the 61 OB and 36 NW individuals based on data input from their metabolic profile (Figure 1A). The total amount of variation explained by the model within the groups is 86.3% [R^2^X (cum)] and between the groups is 72.5% [R^2^Y (cum)]. The predictive ability of the model, obtained by cross-validation, is quite high with a Q^2^(cum) of 62.4%, indicating that the discrimination between the groups on the basis of the metabolic profile is fairly good. The OPLS-DA model allows to correctly classify 95.1% (58/61, sensitivity) OB and 91.7% (33/36, specificity) NW individuals.

The OPLS-DA S-line plot shown in Figure 1B visualizes the covariance and correlation coefficient between the variables and the classification score in the model (see caption of Figure 1B for more information). Strongly discriminating variables combine a clear covariance (p[ctr]) with a high absolute value for the correlation coefficient (abs[p(corr)]). The value of 58 variables is significantly different between OB and NW individuals according to the independent samples t test with *post hoc* Benjamini–Hochberg correction (p < 0.027). Of these 58 variables, 21 have a VIP > 0.7 and show a high correlation coefficient with the classification score (absolute p(corr) > 0.5; Table 2). Lipids (measured via protons on saturated and unsaturated carbons of fatty acid chains of triglycerides as well as phospholipids), N-acetyl glycoproteins and lactate are significantly higher, whereas proline, glutamine, histidine, cysteine, α-ketoglutarate, glucose, citrate and cholinated phospholipids are found to be significantly lower in plasma of OB with respect to NW children and adolescents. The biggest change in plasma concentration between OB and NW children is observed for variable number 87 representing lipids and variable number 98 representing lactate (Table 2).

**Table 2.** **Plasma variables that significantly differ between OB and NW children and adolescents.**

**VAR**	**Spectral range (ppm)**	**Assigned metabolite(s)**	**Increased/decreased in OB with respect to NW**	**Relative concentration**		**p-value^†^**	**%-change**	**p(corr)[1]**	**VIP ± cvSE**

				**OB**	**NW**				
19	5.2752–5.2526	D-Glucose	↓	9.41 ± 1.55	10.86 ± 1.17	0.012	-13.3	-0.524	0.97 ± 0.08

21	4.6940–4.6620	D-Glucose	↓	12.84 ± 2.69	15.38 ± 1.93	0.012	-16.5	-0.548	1.29 ± 0.15

51	3.5914–3.5649	D-Glucose	↓	9.48 ± 1.98	11.16 ± 1.02	0.011	-15.1	-0.506	1.03 ± 0.06

53	3.5510–3.5360	D-Glucose	↓	4.32 ± 0.94	5.19 ± 0.59	0.010	-16.7	-0.537	0.75 ± 0.10

54	3.5360–3.3980	L-Proline + D-Glucose	↓	62.88 ± 13.13	74.96 ± 8.10	0.010	-16.1	-0.560	2.82 ± 0.17

58	3.3230–3.2186	L-Histidine + D-Glucose + -N^+^(C**H**_3_)_3_ of choline head group in SM/PC	↓	61.17 ± 14.22	81.29 ± 7.13	<0.001	-24.8	-0.746	4.28 ± 0.52

62	3.1462–3.1090	L-Cysteine + L-Histidine	↓	2.36 ± 0.60	3.10 ± 0.39	0.002	-23.9	-0.655	0.78 ± 0.21

63	3.1090–3.0860	L-Cysteine	↓	2.48 ± 0.53	3.14 ± 0.39	0.003	-20.9	-0.639	0.72 ± 0.06

64	3.0860–3.0716	L-Cysteine	↓	3.27 ± 0.73	4.03 ± 0.47	0.007	-18.9	-0.579	0.74 ± 0.10

66	3.0640–2.9950	L-Cysteine + α-ketoglutarate	↓	14.05 ± 3.15	17.26 ± 1.91	0.007	-18.6	-0.559	1.52 ± 0.14

67	2.9950–2.8860	α-ketoglutarate	↓	10.15 ± 2.49	12.84 ± 1.68	0.006	-21.0	-0.579	1.41 ± 0.25

73	2.5960–2.5340	Citrate	↓	1.86 ± 0.52	2.56 ± 0.48	0.005	-27.1	-0.669	0.75 ± 0.19

76	2.4920–2.4500	L-Glutamine + α-ketoglutarate	↓	5.71 ± 1.33	7.34 ± 0.98	0.006	-22.1	-0.606	1.13 ± 0.17

86	2.3040–2.2915	Lipids: -C**H**_2_-C=O and -C**H**_2_-CH=CH- in fatty acid chain	↑	2.76 ± 0.81	1.78 ± 0.35	0.001	55.3	0.659	0.91 ± 0.10

87	2.2915–2.2690	Lipids: -C**H**_2_-C=O and -C**H**_2_-CH=CH- in fatty acid chain	↑	6.32 ± 2.45	3.65 ± 1.07	0.002	73.3	0.625	1.44 ± 0.08

88	2.2690–2.2300	Lipids: -C**H**_2_-C=O and -C**H**_2_-CH=CH- in fatty acid chain	↑	4.76 ± 1.54	3.20 ± 0.76	0.004	48.8	0.603	1.07 ± 0.17

91	2.1230–1.9720	Lipids: -C**H**_2_-CH=CH- in fatty acid chain + *N-acetyl signal of glycoproteins	↑	69.70 ± 3.77	63.39 ± 3.54	0.001	10.0	0.813	2.42 ± 0.37

95	1.6860–1.5600	Lipids: -C**H**_2_-CH_2_-C=O and -C**H**_2_-CH_2_-CH=CH in fatty acid chain	↑	16.00 ± 5.24	10.62 ± 2.36	0.003	50.6	0.605	2.00 ± 0.20

98	1.4200–1.3740	L-Lactate	↑	20.66 ± 7.92	12.88 ± 3.47	0.005	60.4	0.554	2.36 ± 0.26

99	1.3740–1.3450	L-Lactate	↑	62.18 ± 16.11	47.06 ± 9.93	0.009	32.1	0.520	3.18 ± 0.26

100	1.3450–1.2458	Lipids: CH_3_-(C**H**_2_)_n_- in fatty acid chain	↑	175.83 ± 41.47	141.12 ± 21.10	0.011	24.6	0.512	4.65 ± 0.47

^†^Benjamini–Hochberg adjusted p-value, calculated using the independent samples t test.

% change is the increase (+) or decrease (−) of the mean in the OB group with respect to the NW group.

CvSE: Standard error of cross-validation; NW: Normal-weight; OB: Overweight or obese; p(corr)[1]: Correlation scaled loading; PC: Phosphatidylcholine; SM: Sphingomyelin; VAR: Variable; VIP: Variable influence on projection.

### Plasma metabolic profile of MHO versus MUO children & adolescents

The OB study group is further subdivided into 18 MHO and 17 MUO children and adolescents (Table 3). Obese participants who cannot be defined as MHO or MUO are classified as metabolically *at-risk* obese (n = 21) or have missing data (n = 9) and are not included in the statistical analysis. Children aged 8–10 years were also classified by the International Diabetes Federation criteria, but a re-examination without the data pertaining to 8–10-year olds – only one subject in both the MHO and MUO group – did not change the study outcome. No statistically significant differences are found for age, gender, pubertal stage, BMI, and BMI SDS between MHO and MUO children. Total cholesterol, LDL-cholesterol, hemoglobin A1c and uric acid did not correlate with the MUO phenotype (data not shown).

**Table 3.** **Characteristics of the obese children and adolescents classified according to metabolic health status.**

**Characteristics**	**MHO (n = 18)**	**MUO (n = 17)**	**p-value**
Age, years	13.4 ± 2.0	13.2 ± 2.0	0.673

Gender, n (%) Male Female	10 (55.6) 8 (44.4)	10 (58.8) 7 (41.2)	1.000

Pubertal stage, n (%) I (prepubertal) II–IV (pubertal) V (postpubertal)	4 (22.2) 9 (50.0) 5 (27.8)	2 (11.8) 7 (41.2) 8 (47.0)	0.494

Weight, kg	80.7 ± 14.5	87.0 ± 20.1	0.295

Height, cm	161.3 ± 9.9	159.7 ± 9.5	0.639

BMI, kg/m^2^	31.4 (23.6–36.0)	33.4 (27.3–44.1)	0.099

BMI SDS	2.7 (1.7–3.2)	2.9 (2.5–3.7)	0.075

SBP, mm Hg	114 ± 10	128 ± 9	<0.001

DBP, mm Hg	69 ± 8	79 ± 6	<0.001

Fasting glucose, mg/dl	92 ± 4	98 ± 7	0.009

HDL-C, mg/dl	47 (40–70)	39 (32–49)	<0.001

Triglycerides, mg/dl	78 (33–149)	134 (58–361)	0.003

Continuous variables are presented as mean ± SD or median (range) and nominal or ordinal scale variables as a number with percentage (%). Metabolically at-risk obese (n = 21) and participants with missing data (n = 9) were not included for statistical analyses.

DBP: Diastolic blood pressure; HDL-C: High-density lipoprotein cholesterol; MHO: Metabolically healthy obese; MUO: Metabolically unhealthy obese; SBP: Systolic blood pressure; SDS: Standard deviation score.

**Figure 2.**
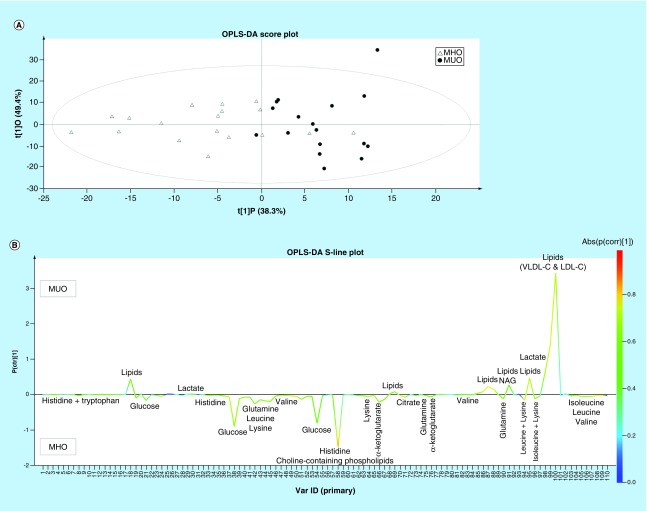
**Discrimination between MHO and MUO based on the ^1^H-NMR-derived metabolic phenotype of blood plasma.** OPLS-DA score plot **(A)** and S-line plot **(B)** obtained for the MHO (△) and MUO (•) children and adolescents. Each participant is represented by its metabolic profile and visualized as a single symbol of which the location is determined by the contributions of the 110 variables in the ^1^H NMR spectrum. The OPLS-DA score plot (LV = 2) shows the first predictive component (t[1]P: 38.3%), explaining the variation between the groups, versus the first orthogonal component (t[1]O: 49.4%) that explains the variation within the groups. The OPLS-DA S-line plot visualizes differences between MHO (negative) and MUO (positive) subjects. The left y-axis represents p(ctr)[1], the covariance between a variable and the classification score. The right y-axis presents p(corr)[1], the correlation coefficient between a variable and the classification score, giving a linear indication of the strength of the correlation. The red color stands for the highest absolute value of the correlation coefficient. Strongly discriminating variables have a large intensity and large reliability. MHO: Metabolically healthy obese; MUO: Metabolically unhealthy obese; OPLS-DA: Orthogonal partial least square discriminant analysis.

Regarding the metabolic profiling, no outliers were detected, and the PCA score plot shows a clear differentiation between clusters of MHO and MUO individuals. Moreover, the PCA model is not confounded by age, gender, pubertal stage and BMI SDS (data not shown). An OPLS-DA model (statistical classifier) trained to differentiate between the 18 MHO and 17 MUO individuals (Figure 2A) is able to explain a total amount of variation within the groups of 87.7% [R^2^X (cum)] and between the groups of 50.5% [R^2^Y (cum)]. The model allows to classify 94.1% MUO individuals (16/17; sensitivity) and 88.9% MHO individuals (16/18; specificity) correctly but still has a mediocre predictive ability of 24.9% [Q^2^ (cum)], most probably due to the limited number of participants.

The OPLS-DA S-line plot shown in Figure 2B visualizes the covariance and correlation coefficient between the variables and the classification score in the model (see caption of Figure 2B for more information). Strongly discriminating variables combine a clear covariance with a high absolute value for the correlation coefficient. Up to 23 variables differ significantly between MHO and MUO individuals according to the independent samples t test with *post hoc* Benjamini–Hochberg correction (p < 0.011). Of these 23 variables, nine have a VIP > 0.7 and show a high correlation coefficient with the classification score (absolute p(corr) > 0.5; Table 4). Lipids and lactate levels are significantly decreased, whereas glutamine, histidine, lysine, valine, isoleucine, leucine and cholinated phospholipid concentrations are higher in plasma of MHO with respect to MUO children and adolescents. The largest difference between MHO and MUO children and adolescents is found for variable number 58 representing cholinated phospholipids and variable number 98 representing lactate (Table 4).

**Table 4.** **Plasma variables that significantly differ between MHO and MUO children and adolescents.**

**VAR**	**Spectral range (ppm)**	**Assigned metabolite(s)**	**Increased/decreased in MHO with respect to MUO**	**Relative concentration**				**p(corr)[1]**	**VIP ± cvSE**

				**MHO**	**MUO**	**p-value^†^**	**%-change**		
37	3.9810–3.9590	L-Histidine	↑	3.98 ± 0.76	3.11 ± 0.87	0.008	27.8	-0.680	0.74 ± 0.25

45	3.7141–3.6680	L-Isoleucine	↑	13.86 ± 3.01	10.77 ± 2.54	0.006	28.7	-0.571	1.42 ± 0.60

58	3.3230–3.2186	L-Histidine + -N^+^(C**H**_3_)_3_ of choline head group in SM/PC	↑	69.24 ± 14.59	50.11 ± 13.01	0.002	38.2	-0.820	3.82 ± 1.01

88	2.2690–2.2300	Lipids: -C**H**_2_-C=O and -C**H**_2_-CH=CH- in fatty acid chain	↓	4.14 ± 1.62	5.79 ± 1.56	0.008	-28.5	0.729	1.01 ± 0.24

90	2.1970–2.1230	L-Glutamine	↑	12.50 ± 1.72	10.87 ± 2.15	0.010	14.9	-0.558	0.92 ± 0.44

94	1.8060–1.6860	L-Lysine + L-Leucine	↑	10.15 ± 1.71	7.96 ± 2.11	0.009	27.5	-0.696	1.21 ± 0.31

98	1.4200–1.3740	L-Lactate	↓	17.35 ± 5.99	25.65 ± 9.75	0.004	-32.4	0.647	2.27 ± 0.38

99	1.3740–1.3450	L-Lactate	↓	56.08 ± 13.45	72.89 ± 18.04	0.001	-23.1	0.706	3.26 ± 0.52

105	1.0400–1.0220	L-Valine + L-Isoleucine	↑	3.59 ± 0.53	2.81 ± 0.75	0.010	27.8	-0.643	0.74 ± 0.22

^†^Benjamini–Hochberg adjusted p-value, calculated using the independent samples t test.

%-change is the increase (+) or decrease (-) of the mean in the MHO group with respect to the MUO group.

CvSE: Standard error of cross-validation; MHO: Metabolically healthy obese; MUO: Metabolically unhealthy obese; p(corr)[1]: Correlation scaled loading; PC: Phosphatidylcholine; SM: Sphingomyelin; VAR: Variable; VIP: Variable influence on projection.

## Discussion

Metabolomics is an emerging field that has been making progress in the area of obesity, and studies have explored potential biomarkers and underlying biological mechanisms of obesity and its cardiometabolic correlates [9,28]. However, the application of NMR-based metabolomics on the obese pediatric population is almost unexplored. This study demonstrates that the plasma metabolic profile of obese and normal-weight children can be clearly distinguished by means of untargeted NMR-based metabolomics. The obese plasma metabolome is characterized by higher concentrations of lipids (triglycerides and phospholipids), N-acetyl glycoproteins and lactate, and lower concentrations of several amino acids (proline, glutamine, histidine, and cysteine), α-ketoglutarate, glucose, citrate and cholinated phospholipids as compared with the normal-weight plasma metabolome.

An MS-based metabolomics study has previously also shown that serum concentrations of glutamine and proline are lower in obese children with respect to their normal-weight counterparts [13]. In addition, obese children showed increased levels of glutamine after substantial weight loss [29]. In this study, we confirm the presence of lower concentrations of glutamine and proline in plasma of obese children. Although the exact mechanism behind reduced proline levels in obesity has not been unraveled yet, lower levels of glutamine could point to an obesity-associated activation of the hexosamine pathway [30]. This hypothesis is further substantiated by lower levels of glucose (increased glucose consumption) and higher levels of N-acetyl glycoproteins found in the plasma of obese children. This is fully in agreement with an NMR-based study on Ningxiang growing pigs that also showed lower levels of glucose combined with higher levels of N-acetyl glycoproteins in the serum of obese compared with lean pigs [31]. The activation of the hexosamine pathway is associated with insulin resistance in skeletal muscles [32], deterioration of pancreatic β-cell function [33] and a reduction in energy expenditure [34]. Altogether, these findings suggest that glutamine might be an early marker for the hexosamine pathway activation in obesity.

Higher levels of N-acetyl glycoproteins may also indicate an increased production of acute phase α-1 acid glycoproteins by the liver in response to proinflammatory cytokines [35,36]. It has been previously suggested that inflammatory mechanisms linking obesity to metabolic and cardiovascular complications are already activated in childhood [37].

Perturbations in lipid metabolism is a typical symptom of childhood obesity [38]. Plasma levels of lipids (saturated and unsaturated fatty acid chains of triglycerides and phospholipids) are increased in obese compared with normal-weight children. Variable number 100 mainly represents the CH_2_ protons of saturated fatty acid chains, in particular of very-low-density lipoprotein (VLDL) and LDL [39]. Increased levels of VLDL and LDL point to an increased hepatic production and secretion of triglyceride containing VLDL particles in the blood which are subsequently converted to LDL [30]. Cali *et al*. showed that this proatherogenic phenotype is strongly related to the intrahepatic lipid content in obese adolescents with normal glucose tolerance [40]. The strong NMR signal corresponding to variable number 58 is representing the nine protons of the three methyl groups of the choline head group of both sphingomyelins and phosphatidylcholines (referred to as cholinated phospholipids). The signal intensity of these cholinated phospholipids is lower in the plasma of obese children as compared with normal-weight children. Cholinated phospholipids are components of the cell membrane and a decreased concentration has been associated with membrane vulnerability to inflammation, which may contribute to the development of obesity and the metabolic syndrome [41]. Moreover, confirmation can be found in an MS-based study revealing increased plasma levels of phosphatidylcholine for obese children and adolescents after a weight loss intervention [29].

Reduced levels of α-ketoglutarate and citrate in the obese plasma can be linked to an impaired tricarboxylic acid cycle; in other words, disturbed energy metabolism [30].

Metabolomic studies aiming to explore the MHO phenotype are still scarce, especially in the pediatric population. Early screening for the MHO phenotype could support the development of effective prevention and treatment strategies in the fight against childhood obesity. Investigating the two metabolic phenotypes of obesity reveal that the MUO phenotype has lower plasma levels of glutamine and histidine as compared with the MHO phenotype. This points to an obesity-associated activation of the hexosamine pathway and the presence of inflammation, respectively [30,42]. Nowadays, plasma concentrations of branched-chain amino acids (BCAAs; valine, isoleucine and leucine) are increasingly being investigated in the context of obesity, diabetes and cardiovascular disease. However, previous study findings on BCAAs – both in obese children and adults – are inconsistent. In our study, BCAAs are found to be significantly reduced in plasma of MUO as compared with MHO children and adolescents. In line with our findings, Mihalik *et al*. found that adolescents with Type 2 diabetes showed lower concentrations of BCAAs as compared with obese as well as normal-weight individuals and suggested that these lower concentrations result from an increased gluconeogenic drive [12]. In adults, however, higher circulating levels of BCAAs have been correlated with insulin resistance and future development of diabetes [43]. This finding most likely indicates different metabolic mechanisms for children and adolescents as compared with adults.

## Conclusion

The presented ^1^H NMR study reveals that the obese plasma metabolome is clearly different from the normal-weight plasma metabolome. Obese children show higher concentrations of lipids, indicating that the hepatic production and secretion of triglycerides is likely increased. They further show lower levels of glutamine and glucose, and higher levels of N-acetyl glycoprotein which could point to an activation of the hexosamine pathway. Reduced levels of α-ketoglutarate and citrate in the obese plasma can be linked to an impaired tricarboxylic acid cycle, reflecting a disturbed energy metabolism. Lower levels of cholinated phospholipids, which were observed for obese as well as MUO children, have been suggested previously to be associated with the development of obesity and the metabolic syndrome. Last but not least, BCAAs (valine, isoleucine and leucine) were found to be lower in the plasma of MUO as compared with MHO children which might point to an increased gluconeogenic drive, as typically observed in diabetes. To conclude, this study reveals new valuable findings in the field of metabolomics and childhood obesity. Research is ongoing to confirm and to validate the observations in an independent cohort. A better understanding of childhood obesity will be of great importance toward the development of more personalized prevention and treatment in future.

## Future perspective

Knowledge from this study will aid in a greater understanding of metabolic derangements in obese children. Moreover, this study adds to the awareness that metabolic phenotypes within obesity exist (already in youth) and can support the development of future personalized healthcare. In this way, the plasma ^1^H NMR metabolic profile may become useful in identifying obese children at greatest risk for developing metabolic diseases later in life. Although NMR is currently overshadowed by MS and is not yet applicable in routine clinical labs, the importance of NMR is increasingly being recognized. NMR spectroscopy is highly reproducible and quantitative over a wide dynamic range; gives insight in metabolic pathways and fluxes by using isotope labels; allows easy, highly reproducible and fast sample preparation (no extractions); and is nondestructive. The use of higher-field ^1^H NMR spectroscopy (also in combination with MS), new statistical models and an integrative approach to analyze gene-transcriptome-proteome-metabolite data will add to a better and more complete understanding of metabolic derangements in childhood obesity. Further longitudinal cohort studies in children and adolescents are needed to confirm these fundamental findings and to assure validity. The inclusion of other biofluids such as urine, salivary or feces can be of added value for pediatric research, especially since the procedures of collection are noninvasive. Also, including factors such as nutrition, level of physical activity and gut microbiota composition and function will add great value to this research. Relevant biomarkers for the assessment of obesity-related metabolic diseases can be discovered and this might open new perspectives to develop more targeted and personalized clinical management of childhood obesity. This will reduce healthcare costs and result in a better life expectancy and quality of life for the current and next generations of children and adolescents.

**Summary points**

- The plasma metabolic profile of obese children can be clearly differentiated from that of normal-weight.
- Proton NMR spectroscopy-based metabolic profiling of plasma shows higher concentrations of lipids (triglycerides and phospholipids), N-acetyl glycoproteins and lactate, and lower concentrations of several amino acids (proline, glutamine, histidine and cysteine), α-ketoglutarate, glucose, citrate and cholinated phospholipids in obese compared with normal-weight children.
- Metabolites that significantly differ between obese and normal-weight children might point toward an activation of the hexosamine pathway, a proinflammatory state, an impaired tricarboxylic acid cycle and perturbations in lipid and cholinated phospholipid metabolism in childhood obesity.
- Metabolically healthy obese children show increased plasma levels of branched-chain amino acids (valine, isoleucine, and leucine) as compared with their unhealthy counterparts which might point to an increased gluconeogenic drive, as typically observed in diabetes.
