# Supplementary figures and images for: Identification of metabolic phenotypes in childhood obesity by 1H NMR metabolomics of blood plasma
Source: Future Sci OA. 2018 May 23;4(6):FSO310. doi: 10.4155/fsoa-2017-0146 (PMC6060399; doi:10.4155/fsoa-2017-0146)

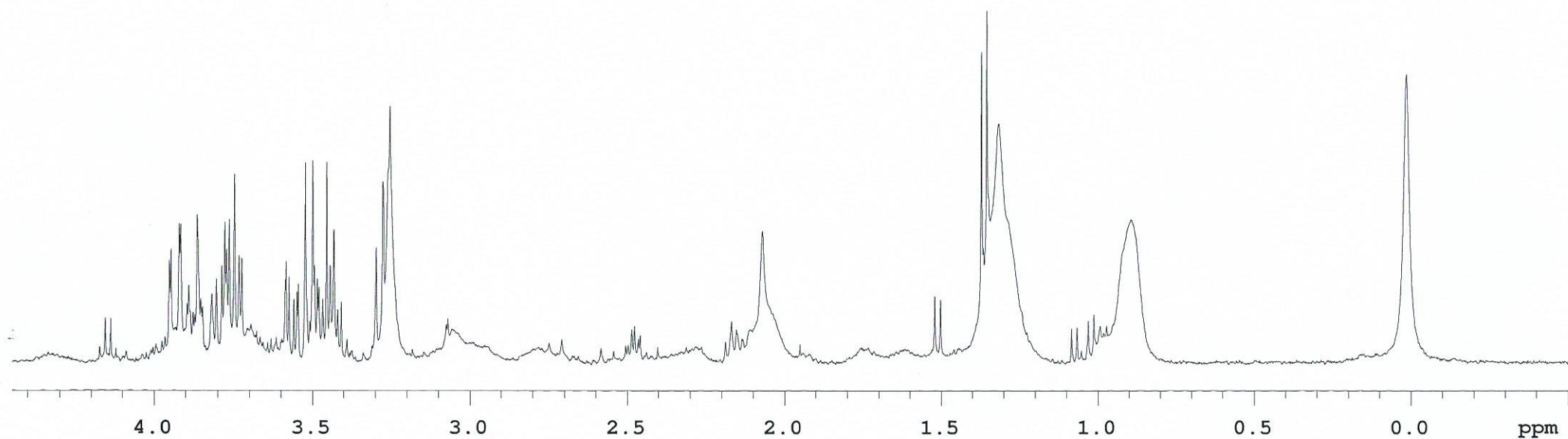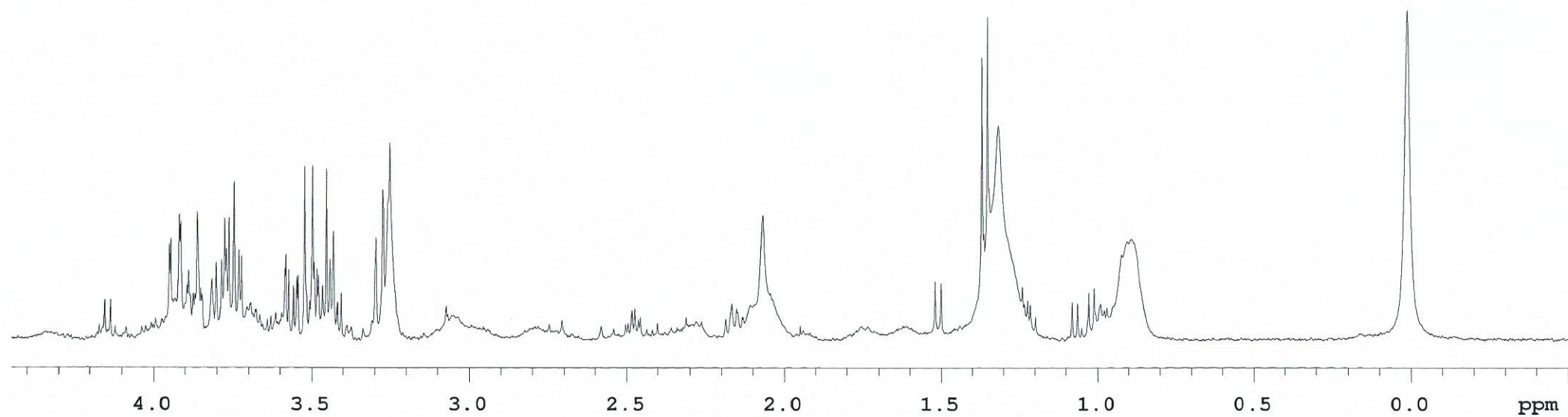

Supplement: Supplementary file 1 [file fsoa-04-310-s1.pdf]
