# Supplementary material for: Identification of metabolic phenotypes in childhood obesity by 1H NMR metabolomics of blood plasma
Source: Future Sci OA. 2018 May 23;4(6):FSO310. doi: 10.4155/fsoa-2017-0146 (PMC6060399; doi:10.4155/fsoa-2017-0146)
Supplement: Supplementary file 2 [file fsoa-04-310-s2.pdf]

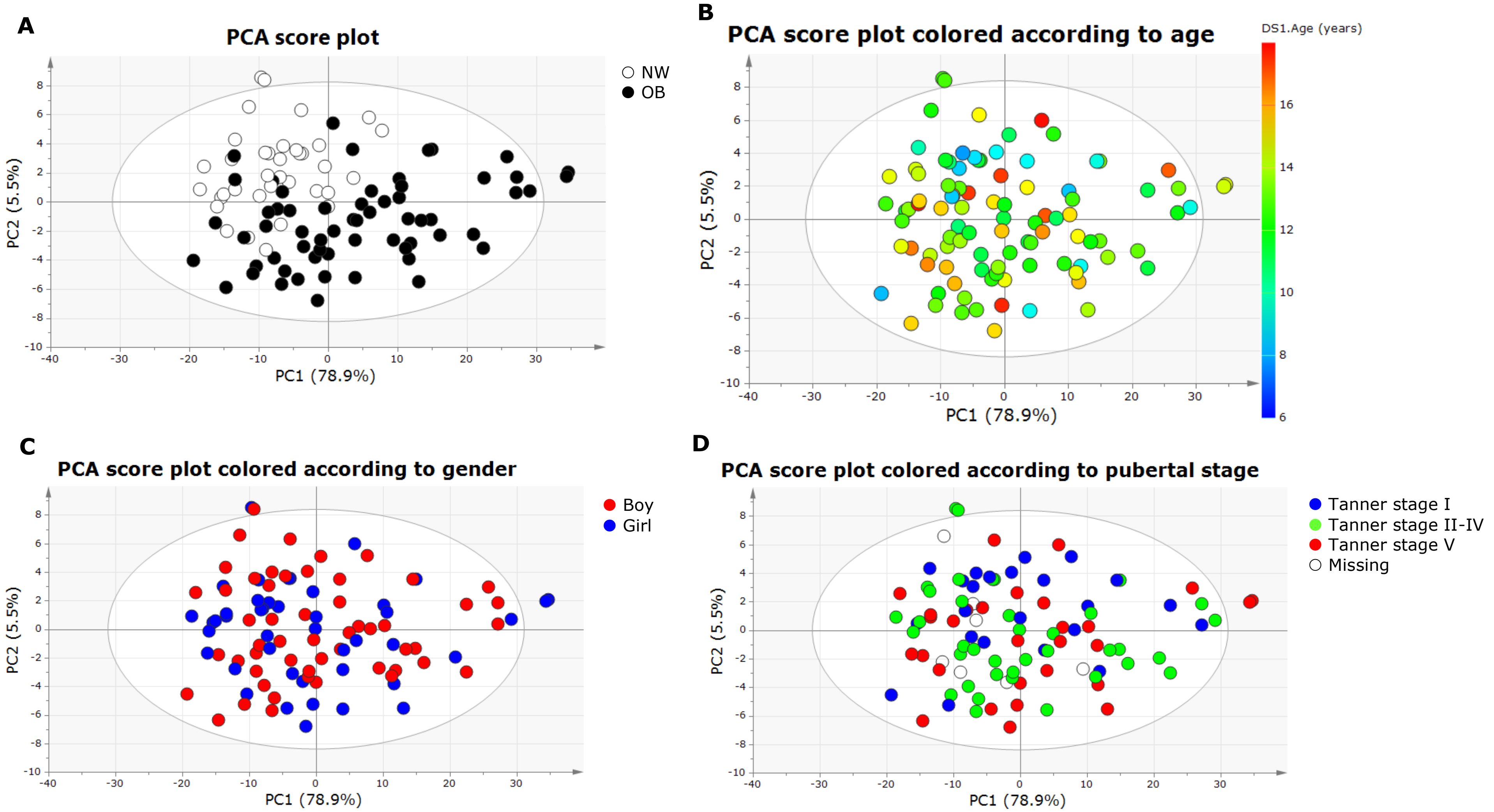

Figure S2 PCA score plots based on the 110 plasma ^{1}H-NMR variables of 61 OB and 36 NW children colored according to weight status (A), age (B), gender (C), pubertal stage (D). Each participant is represented by its metabolic profile and visualized as a single symbol of which the location is determined by the contributions of the 110 variables in the ^{1}H-NMR spectrum. The PCA plot shows the first principal component (PC1: 78.9%), explaining the largest variance within the dataset, versus the second principal component (PC2: 5.5) that explains the second largest variation within the data. The PCA score plot of age (B) is continuously colored according to an age between 8 and 18 yrs.
